# Bioprocessing and the Production of Antiviral Biologics in the Prevention and Treatment of Viral Infectious Disease

**DOI:** 10.3390/vaccines11050992

**Published:** 2023-05-17

**Authors:** Elaine Meade, Neil Rowan, Mary Garvey

**Affiliations:** 1 Department of Life Science, Atlantic Technological University, F91 YW50 Sligo, Ireland; 2 Centre for Precision Engineering, Materials and Manufacturing Research (PEM), Atlantic Technological University, F91 YW50 Sligo, Ireland; 3 Bioscience Research Institute, Technical University Shannon Midlands Midwest, N37 HD68 Athlone, Ireland

**Keywords:** antiviral, vaccines, cytokines, expression systems, pandemic

## Abstract

Emerging, re-emerging and zoonotic viral pathogens represent a serious threat to human health, resulting in morbidity, mortality and potentially economic instability at a global scale. Certainly, the recent emergence of the novel SARS-CoV-2 virus (and its variants) highlighted the impact of such pathogens, with the pandemic creating unprecedented and continued demands for the accelerated production of antiviral therapeutics. With limited effective small molecule therapies available for metaphylaxis, vaccination programs have been the mainstay against virulent viral species. Traditional vaccines remain highly effective at providing high antibody titres, but are, however, slow to manufacture in times of emergency. The limitations of traditional vaccine modalities may be overcome by novel strategies, as outlined herein. To prevent future disease outbreaks, paradigm shift changes in manufacturing and distribution are necessary to advance the production of vaccines, monoclonal antibodies, cytokines and other antiviral therapies. Accelerated paths for antivirals have been made possible due to advances in bioprocessing, leading to the production of novel antiviral agents. This review outlines the role of bioprocessing in the production of biologics and advances in mitigating viral infectious disease. In an era of emerging viral diseases and the proliferation of antimicrobial resistance, this review provides insight into a significant method of antiviral agent production which is key to protecting public health.

## 1. Introduction

Communicable disease are caused by microbial infectious agents, including bacteria and viruses, which spread within populations via direct and indirect contact. Viral communicable diseases have come into the spotlight in recent years due to the COVID-19 pandemic highlighting the impact such pathogens can cause. Additional novel viral outbreaks occurring in recent decades include Ebola [1], severe acute respiratory syndrome (SARS) [2], Middle East respiratory syndrome (MERS), Zika, and Chikungunya [1]. Monkeypox, a zoonotic viral disease caused by the monkeypox virus (MPXV), has resulted >85,000 confirmed MPXV cases and >89 deaths worldwide since January 2022 [2]; it was declared a public health emergency by the WHO in July 2022 [3]. Furthermore, more recently, the Marburg virus [4,5] has emerged.

The interaction between animals, humans and the environment is now considered under the One Health approach to mitigating infectious disease. One Health is an important concept, as approximately 80% of human viral pathogens are zoonotic with climatic, agricultural and anthropological factors contributing to disease outbreak [4]. Furthermore, zoonotic viruses regularly result in spillover events in livestock animals which act as reservoirs for human exposure [5]. The studies of Cui et al. (2023) have identified the bat as a reservoir for the highest diversity of coronaviruses, including SARS, MERS and SARS-CoV-2 [6]. Viral adaptability resultant from genetic mutation, recombination and reassortment allows them to adapt to new hosts and ecological niches [7]. The high mutation rate observed in ribonucleic acid (RNA) viruses in particular allows them to evolve rapidly, adapting to environmental and hosts niches [8]. Zoonotic diseases have a greater economic impact and higher rate of mortality than non-zoonotic viruses [8].

Undoubtedly, public awareness and dissemination of information relating to viral disease risk factors and prevention measures is essential in preventing epidemic- and pandemic-scale outbreaks. The application of vaccines which prevent respiratory viral hospitalizations due to influenza, pneumococcal disease (PD), COVID-19 and a hypothetical Respiratory Syncytial Virus (RSV) was determined to save ca. GDP 45 million in the United Kingdom alone [9]. Vaccination remains the best prophylactic tool for viral disease prevention. Antigenic shift and antigenic drift, however, allow for viral adaption to overcome innate host immunity and acquired vaccine immunity. Small molecules as antiviral therapeutics have been the mainstay in viral therapy for decades. The mode of action of antiviral drugs includes arresting the viral life cycle, interference with the viral genome, entry inhibitors, integrase inhibitors, protease inhibitors and immunomodulators [10]. Issues arise with host cell toxicity, particularly for combination therapies, with resistance to antiviral therapy observed in the treatment of AIDS, hepatitis B and C, herpes and influenza [11]. However, advances in bioprocessing and recombinant DNA technologies have allowed for advances in the production of vaccines and biologicals, showing great efficacy and safety [12]. As such, there is a move towards the use of biologics such as mAbs, interferons and nucleic acid therapies in the mitigation of viral disease. This review outlines the role of bioprocessing in the production of biologics and advances in mitigating viral infectious disease. A literature search was completed using relevant search engines (PubMed, Science Direct, etc.) within a 10-year timeframe, with the focus on most recent articles applicable to novel antiviral strategies.

## 2. Host Response to Viral Infection

At the host level, viral infectious disease is mitigated via an innate and acquired immune response. Upon entry to the body, the immune system recognizes viruses via activation of the pattern recognition receptors (PRRs), leading to autophagy and the production of cytokines and interferons (IFNs) [13]. PRRs interact with pathogen-associated molecular patterns (PAMPs) such as Toll-like receptors (TLRs), RIG-like receptors (RLRs), the cyclic gMP-AMP synthase (cGAS) and the IFN-γ-inducible protein 16 (IFI16) [14]. IFN-1 is particularly important in viral infection as it activates natural killer (NK) cells which can kill host infected cells and stimulate release of proinflammatory cytokines, including interleukins (ILs) [15]. Based on receptor association, there are three recognized classes of IF: types I, II and III [16]. Type I are considered viral IFs and include IFN-α, IFN-β, IFN-ω and IFN-τ, with type II IFN being IFN-γ [17]. More recently, the type III interferon (IFN-λ) was found to play a key role in immune responses to microbial species, including viruses [18]. Upon binding to their respective IF receptors, IFN-α and IFN-β induce the expression of genes referred to as interferon stimulated genes, which inhibit viral protein synthesis and promote the expression of major histocompatibility complex (MHC) on cell surfaces, promoting the adaptive immune system, specifically cell-medicated immunity [19]. IF-stimulated genes include protein kinase R, which inhibits viral translation and protein synthesis. The adaptive immune response targets both the virus and the virally infected cell via humoral immune and cell-mediated response pathways. Antibodies (ABs) and the complement system are the mechanisms of humoral response. ABs bind the virion, preventing entry into host cells and consequent viral replication. The complement system is active in both innate and adaptive immunity, having intra- and extracellular activity at all stages of viral infection [20]. This system dipslays antiviral activity via opsonization, membrane attack complex formation on virions and infected cells, proteasomal degradation action, regulation of chemotaxis and promotion of inflammation [21]. However, viruses can evade IF activity by downregulating IF receptor expression, inducing regulators and suppressing cytokine signalling [16]. Viruses also evade host immunity due to their heterogeneous nature and antigenic drift and shift. RNA viruses typically have high mutation rates, which generate viral variants capable of avoiding the immune response, whereas deoxyribonucleic acid (DNA) viruses have large genomes coding for many proteins involved in avoidance of host immunity [19]. At the community level, endemic and pandemic outbreaks are prevented prophylactically by mass vaccination programmes with metaphylactic treatment primarily reliant on small molecule antivirals. More recently, bioprocessing has been employed to produce interferon and antibody biologics for the treatment of viral infectious disease.

### 2.1. Traditional Production of Vaccines

Traditional methods of producing vaccines rely on the use of dead or weakened pathogenic microbes or pathogen-produced toxins or proteins [22], which generate an immune response within the vaccinated individual. Vaccine types can be categorized as those that deliver a specific target antigen, e.g., viral glycoprotein, or those that deliver the entire pathogen in an inert or weakened form, i.e., inactivated or live-attenuated virus [1]. Initially, attenuation was attempted by passage in abnormal hosts such as chicken embryos for polio and rabies viruses. However, cell culture has led to passage in vitro using cell lines as viral growth platforms. Attenuated vaccines for polio, measles, rubella, mumps and varicella have been developed by cell-culture passage [23]. Other methods of producing attenuated vaccines have been developed, including codon targeting and producing auxotrophic strains by deleting or silencing a gene [22]. The studies of Fang et al. (2023) describe the production of an attenuated Dengue strain via deletion of amino acid residues associated with the site of N-glycosylation [24]. In terms of immunity, live attenuated vaccines generate the best immune response, stimulating all components of the immune system as the live viral pathogen in the absence of morbidity and mortality [25]. The recent research of Deng et al. (2023) describes the in vivo application of a live attenuated nasal vaccine against COVID-19 in test mice and hamsters, where high levels of antibodies and T-cell activation occurred in mice [26]. Such vaccines, however, have issues with reversion to highly pathogenic forms, thus inducing morbidity/mortality; as such they cannot be implemented for highly virulent species [1]. Live vaccines have the potential to replicate uncontrollably in immunocompromised persons, further restricting their use; inactivated vaccines pose no such risk [27]. Inactivated vaccines are non-live vaccines produced via heat and chemical exposure. These vaccines induce immunity toward a killed/inactivated virus (unlike live attenuated vaccines) and are not capable of reversion to wild-type virulent strains. Chemicals used for inactivation include ascorbic acid (rabies vaccine), psoralen (dengue vaccine), ethylenimine, formaldehyde and -propiolactone [22]. Ultraviolet (UV) exposure and gamma irradiation are also used to produce influenza A vaccine, and heat inactivation is utilized for polio vaccine [28]. The JE virus, for example, is cultured in monkey kidney epithelial cells (Vero cells) in vitro and inactivated using formalin [29]. The antigenic component of such inactivated vaccines consists of a killed whole organism (inactivated polio vaccine) or purified proteins from the organism or recombinant proteins (hepatitis B virus vaccine) [27]. Some viral vaccines consist of purified proteins, e.g., influenza vaccines are produced by growing the virus in embryonated eggs followed by degradation using detergents to obtain the antigenic viral hemagglutinin (HA) protein [23]. For such vaccines (subunit and conjugate vaccines), segments of the pathogen are administered to generate the immune response. However, inactivated vaccines or subunit vaccines often do not produce a strong immune response or a cell-mediated response, e.g., cytolytic T cells (CTLs), which is needed for certain viral disease [25]. CTLs are considered an important part of the immune response as they target the virally infected cells and epitopes that are conserved between different viral strains, including internal and functional proteins which are not targeted by humoral ABs [25]. Adjuvants such as insoluble aluminium salts, liposome-based adjuvants and oil-in-water emulsions such as MF59, AS01, AS04, AS03 and cytosine phosphoguanosine (CpG) 1018 are added to boost the strength and durability of the immune reaction to the vaccine [30]. The addition of adjuvants to vaccine formulations possesses immunogenicity and biocompatibility issues. Furthermore, traditional vaccine production methods are extremely time-consuming, needing extensive periods for development and extensive clinical safety and efficacy testing prior to use. With intense regulatory approval, large-scale production and quality control protocols generate further time constraints [1]. Consequently, in emergency situations such as pandemic-scale events they do not allow for a swift means of public health protection against rapidly emerging viral disease. Indeed, the COVID-19 pandemic highlighted the urgent need for vaccine production platforms which are fast, flexible and amenable to upscaling at industrial levels [27]. Genetic engineering and advances in recombinant DNA (RDNA) technology and bioprocessing have encouraged novel vaccine production methods.

### 2.2. The Role of Bioprocessing

Bioprocessing and the growth of genetically modified (GM) cells expressing heterologous proteins has greatly impacted the production of therapeutic biologics. Bioprocessing involves the growth of selected cells in fermentation tanks/bioreactors under controlled conditions optimal for cell growth and protein expression. Eukaryotic cell lines (CHO cells, human cells, insect cells), eukaryotic microbial cells, e.g., *Saccharomyces cerevisiae* and *Pichia pastoris* (*Komagataella phaffii*) and prokaryotic cells, e.g., *Escherichia coli* have become invaluable expression systems for the production of many recombinant proteins [31]. Prokaryotic systems and eukaryotic yeast and fungal systems have many advantages, including ease of GM, ease of scale-up, rapid growth rate and cheap growth requirements. Mammalian cell lines, however, are more expensive to culture and are prone to microbial contamination, particularly viral contamination [32]. Proteins produced by mammalian cells are more accurately folded and subject to post-translational modifications (PTMs), which are absent in prokaryotic systems, where bacterial toxins also represent an issue [33]. PTM involves any process which alters protein composition and includes the irreversible or reversible addition of a chemical group, e.g., phosphate, carbohydrates (glycosylation) or polypeptides (ubiquitylation); this is related to the biological activity of the protein [34]. PTMs important for vaccine development include glycosylation, acetylation, sulfation, methylation, amidation, SUMOylation, ubiquitylation, lipidation, formylation and phosphorylation [35]. PTMs occurs in eukaryotic cell organelles including the nucleus, cytoplasm, endoplasmic reticulum (ER) and Golgi apparatus, which are absent in prokaryotic cells [36]. Therefore, biologics expressed by bacterial expression systems need in vitro processing and the addition of PTM steps during synthesis, increasing costs and reducing yield [37]. Considerations such as the plasmid used, promoter usage, control of proteolytic degradation, expression rate and location (extracellular, intracellular) of proteins affects the quality and quantity of biologics produced in expression systems, with purification strategies also impacting on the vaccine’s antigenic and immunogenic properties [38]. Bioreactors are operated as batch, fed-batch or continuous systems and with operation considerations including cell type, media composition, substrate concentration, cell density of the biocatalyst, product inhibition, pH, oxygen requirements and temperature, where protein production is typically higher in continuous mode than in batch systems [36]. Most commercial virus production processes utilise Vero, Madin–Darby canine kidney (MDCK), human foetal lung fibroblast cells (MRC-5) and human lung fibroblast (WI-38) continuous cell lines, which maintain an anchorage-dependent growth [39].

### 2.3. Novel Vaccine Approaches

Recent advances in vaccine production have generated protein-based subunit vaccines, virus-like particle (VLP) vaccines, viral vector- and nucleic acid-based (RNA and DNA) vaccine modalities [40]. The production formats of such vaccines aim to lessen the time constraints, issues with reversion to pathogenicity, immunogenicity and biocompatibility issues observed with traditional vaccine types [36]. The use of RDNA technology along with mammalian and non-mammalian expression systems in bioreactors has allowed for cheaper, faster production of more diverse vaccine types including subunit-based and viral-like particle-based vaccines (Table 1) [41].

#### 2.3.1. Viral-like Particles

VLPs are nanoscale structures consisting of viral proteins formulated into a vaccine lacking the viral genetic material, making them non-pathogenic [54]. Such VLPs containing the antigen proteins of viruses are produced in prokaryotic (bacterial) and eukaryotic expression systems including mammal, plant, insect and yeast cells to increase the immune response and durability of subunit vaccines [55]. VLPs conform their protein structure to the natural size and shape of viruses without the genetic material stimulating an immune response and without resulting in morbidity. Additional advantages include the ability to carry immune-modulators which further stimulate the immune response, and the stimulation of both humoral and cell-mediated immunity while being safe for immunocompromised persons [54]. The prokaryotic *E. coli* has many advantages as an expression system, including inexpensive growth requirements, rapid growth rate, high expression levels and ability to be scaled up, and it has successfully been applied for the production of VLP Hecolin [44]. Additionally, an HPV Type 16 L1 VLP has been produced using *Lactobacillus casei* as an expression system [56]. *S. cerevisiae* and *P. pastoris* have been applied for the production of VLPs Engerix-B (HBV vaccine) and Gardasil (HPV vaccine) [54]. Yeast cells are more suited to the production of non-enveloped single and multilayered VLPs; VLPs of human parvovirus B19, adeno-associated virus and human bocavirus have been produced in *S. cerevisiae* [38]. However, yeast-expressed biologics lack the complex PTMs of mammalian cells and are prone to high mannose glycosylation, which can impact on functionality [33]. Numerous mammalian cells can be used for VLP production, including enveloped VLPs, which result in biologics with more suitable PTMs, although the yield is lower [56]. Chinese Hamster Ovary (CHO) cells are an excellent expression system for recombinant protein production and are applied in the production of Hanta virus VLP effective in mice [57]. Additional cell lines used for VLP production include the HEK293 for rabies, human immunodeficiency virus (HIV) and influenza VLPs and CAP-T cells for HIV VLP production [58]. Sang et al. (2023) describe the production of an MRNA vaccine in HEK293T cells for the Monkeypox virus which produced an antibody response in mice [2]. In terms of VLP yield, bacteria and yeast are high-yield systems with 0.75 to 700 µg of protein per ml of culture, animal cell systems achieve yields between 0.2 and 18 µg/mL, whereas yields ranging from 0.018 to 10 µg/mL are possible for mammalian cell systems [56]. Plant expression systems such as lettuce, potatoes and tomatoes have been used for the production of VLPs against Norwalk virus however, yield is low and time consuming [54]. More recently, Health Canada approved the world’s first plant-derived VLP COVID-19 vaccine, Covifenz^®^ (produced using the Nicotiana benthamiana expression system), though the agent has not yet been approved by WHO [48]. Insect expression systems for production of enveloped and non-enveloped VLPs include the baculovirus/insect cell (B/IC) system and cell lines derived from Spodoptera frugiperda (Sf9/Sf21) and Trichoplusia ni (Tn5) [59], e.g., the Cervarix VLP for Human Papilloma virus (HPV) is produced using such systems. VLP vaccines are now produced for hepatitis B virus (HBV) (Engerix), HPV (Cervarix and Gardasil^®^) and HBV Recombivax^®^, with VLPs for influenza, rotavirus, Zika and HIV undergoing clinical trials [55]. Urakami et al. (2017) successfully developed a novel VLP-based vaccine platform utilizing VLPs from the chikungunya virus which initiated an immunogenic response in test animals [60].

#### 2.3.2. Viral Vector Vaccines

Viral vectored vaccines consist of a recombinant virus, which may be capable of replicating, where the genome has been genetically modified to express the antigen of the infectious agent being targeted. The presence of the target antigens on the viral particle stimulates potent humoral and cell-mediated immunity upon administration [27]. Recombinant viral vectors have been used to deliver antigens for decades with adenoviruses, poxvirus, herpesvirus and lentiviruses acting as vaccine vectors [61]. Replication-deficient vectors which cannot self-replicate require the use of promoter regions to express the antigen of interest and often require repeat dosing and/or the addition of adjuvants [61]. Replicating vectors have the clear advantage of better mimicking the immune response, inducing cytokines and other immune mediators to produce a potent response. Viral vectors produce a long-lasting immunity and can be targeted into specific tissues [62]. Adenoviruses, non-enveloped double-stranded DNA viruses, generally cause a mild self-limiting respiratory and ocular infection in humans [62]. As viral vaccine vectors, adenoviruses are a new technology due to their broad tropism. Adenoviruses do not integrate their genome into the host genome, giving them a clear advantage in terms of safety over other vectors such as the lentiviruses [63]. Integration of the viral genome into the host genome is associated with genotoxicity and carcinogenicity. Adenoviral vectors have been implemented to develop vaccines against HIV, Ebola virus disease, SARS-CoV-2 and Zika virus [64,65]. To produce adenoviral vectors, the wild-type adenoviral genome is removed and an expression cassette containing the gene and promoters from the pathogen of interest is added with growth in suitable cell lines to generate the antigen of interest [66]. In bioprocessing, the production process for adenoviruses starts by growing a suitable cell line to optimal cell density, usually in a stirred tank bioreactor for infection with the adenovirus post-inoculation [67]. Cell lines used for the cultivation of adenoviruses include human embryonic kidney cells (HEK 293), MDCK, mouse fibroblast cells (L929) and human lung cells (A549). Due to the high prevalence of these viruses in populations, issues with pre-existing immunity to adenoviruses hinders the activity of adenoviral vectors where unwanted side effects may also occur including hepatotoxicity and systemic inflammation [68]. The use of adenoviruses with lower prevalence rates, the removal of epitopes recognized by PRRs in humans, and use of non-human serotypes can help circumvent these issues [66]. Bovine, porcine, murine and canine adenoviruses have been applied in the production of adenoviral vaccine vectors [69]. Due to their highly immunogenic nature, poxviruses are another viral vector candidate which have been used to produce vaccines against HIV-1 and malaria and can produce multi-antigen vaccines against different pathogens [70]. Poxviral vectors are generated via homologous recombination in cells including kidney epithelial cells, Vero cells and African green monkey-derived cells (BSC-40 cells) [71], and are replication-deficient or rendered deficient in avian cells [72]. Viral vector vaccines are also suitable for many administration routes, including intramuscular, intranasally, orally and intradermally [27]. Oral vaccine delivery has many advantages, including ease of administration, being pain-free, and capable of self-administration with limited undesired effects, as seen in parenteral delivery methods [73].

#### 2.3.3. Nucleic Acid-Based Vaccines

Nucleic acid-based vaccines consist of either DNA (as plasmids controlled by a promoter) or RNA as messenger RNA (mRNA) encoding the target antigen for uptake in host cells, which will induce humoral and cellular immune responses in the host [74]. The gene encoding the antigen which is incorporated into the host cell can produce multiple copies of the immune-stimulating viral antigenic proteins [75]. Nucleic acid vaccines are easy and fast to develop, highly versatile and adaptable to emerging viral pathogens, as seen with the SARS-CoV-2 pathogen [74]. Additionally, due to the degradation process in host cells, mRNA-based vaccines reduce the risk of infection and insertion-induced genetic mutations [76]. mRNA vaccines are also relatively unstable and require cold storage, which is a major limitation impacting storage, distribution and the efficacy of these vaccine types [77]. Upon administration and mRNA-coded antigen production, host dendritic cells (antigen-presenting cells) engulf and process the target antigen, which is responsible for inducing an immune response [78]. The cellular delivery of mRNA to the host cell nucleus is not needed, as transcription is not required [79]. DNA vaccines, however, must be delivered into the cell nucleus where the DNA is transcribed to mRNA, which initiates antigenic protein formation; this may ultimately change the genetic composition of the host cell permanently [80]. DNA vaccines have weaker immunogenicity, but are more stable and capable of long-term storage [76]. A clear disadvantage of nucleic acid-based systems relates to the need to be delivered directly into the host cell where a carrier molecule must be added [74]. Lipid-based delivery systems and polymer-based delivery systems have been developed for vaccine delivery [81]. Lipid-based delivery systems, such as lipid-based nanoparticles (LNPs), demonstrate good biocompatibility to host cells and provide protection against protease degradation of the nucleic acids and achieve endocytosis [82]. Poly-(lactic-co-glycolic acid) (PLGA) and PEG are FDA-approved polymers for vaccine application due to their biocompatibility and biodegradability in vivo [83]. Polymeric carriers have a good nucleic acid loading efficiency, allowing for improved stability while preventing degradation of the nucleic acids [81]. Polymers carrying antiviral agents can improve solubility and prolong the uptake and retention time of the antiviral agent into cells [84]. The addition of adjuvants to the polymer carriers potentially offers a means of increasing the immune response. Conjugation of nucleic acid-based therapeutics is also an area of promising research to improve stability, delivery and uptake [78]. mRNA vaccines are produced by in vitro synthesis, involving enzymatic processes where DNA vaccines are manufactured in bioreactors that grow the bacteria containing the viral genetic information on a plasmid [85], allowing for large-scale manufacturing. DNA vaccines can also be designed to deliver antigen genes and genes which provoke an immune response, including cytokine genes and other immune-stimulating molecules [74,76]. There is a risk, however, of anti-DNA antibodies inducing autoimmune disease in patients, which was observed with the HBV DNA vaccine containing adjuvants [74]. Importantly, nucleic acid vaccine efficacy is impacted by viral mutagenesis and the emergence of mutant viral strains, as observed with the SARS-CoV-2 virus and its mutated variants [86].

#### 2.3.4. Whole Yeast-Based Vaccines

Yeast are known for their excellent ability to express heterologous proteins and have proven useful in vaccine development. Yeast have many advantages as culture organisms for biologics, including fast growth rate, low-cost media and some PTM ability, and they are considered generally recognized as safe (GRAS) by the FDA [36]. Yeast are amenable to large-scale production in bioreactors and are amenable to genetic manipulation and the incorporation of plasmids containing the gene of interest coding the desired biologic. Indeed, yeast expression systems have emerged for the development of vaccines, and yeast as platforms for the production of whole yeast-based vaccines (WYVs) in particular [40]. Yeast cells expressing foreign proteins such as viral antigens can be administered to induce an immune response. The yeast cell membrane containing chitin and glycocalyx can also act as a microencapsulation system which can carry nucleic acids [87]. The cell wall components of yeast chitin, glucan and mannan have adjuvant activity and can act as natural immune stimulators boosting the vaccination [87]. As a non-pathogenic strain, *S. cerevisiae* is easily genetically modified, possess strong adjuvant properties and long-term stability, while allowing for oral delivery due to its resistance to gastric degradation [36]. To date, several antigens including antigens from HBV and porcine epidemic diarrhoea virus were successfully expressed on the yeast surface for the development of oral vaccines [88]. This is important, as the production of vaccines with recombinant HBV antigens has not been successful in inducing humoral and cell-mediated immunity [87]. The influenza H5N1 HA has been displayed on the surface of *S. cerevisiae* via incorporation of an expression plasmid pYD1 with immune response generated in animal models [89]. The activity of whole recombinant *S. cerevisiae* cells expressing foreign antigens activating dendritic cells, antigen-specific cytotoxic T lymphocytes, and conferring protective cell-mediated immunity in animal studies has been demonstrated [90]. Yeast can act as whole yeast-based vaccines regardless of their cell viability [36].

### 2.4. Production of Interferons and Monoclonal Antibodies

As previously outlined, interferons are potent regulators of the humoral and cell-mediated immune response and are highly associated with antiviral activity [15]. Post-viral infection type I IF is expressed with innate antiviral activity inhibiting viral replication and aids in inducing long-term immunity via adaptive immune responses [91]. Therapeutic IFs were initially derived from leukocytes and lymphoblastoid cell lines; RDNA technology, however, has quickly encouraged the use of recombinant IF production in large-scale bioreactors [92]. Many expression systems (Table 2), including *E. coli* and yeast, can be used to produce recombinant cytokines such as interferons [40]. Importantly, unglycosylated IFs are functionally similar to glycosylated Ifs, with the exception of IF β [93]. The recombinant human interferon β lacks glycosylation when produced in *E. coli* [94]. *P. pastoris* and *Y. lipolytica* are yeast expression systems for the production of interferon-α for the treatment of hepatitis B and C [95]. Currently, *E. coli* and *P. pastoris* are the most widely used expression systems for producing clinical IFs; IF β is produced in CHO cells as its activity increases with glycosylation [92]. Proteins produced by *P. pastoris* are secreted intracellularly or extracellularly where protein degradation can be an issue; protein protease-deficient strains, e.g., SMD 1168, can be used to overcome this [96]. Studies report the use of the silkworm baculovirus expression system to produce heterologous proteins type III IF λ where *B. mori* has a high level of protein synthesis with complex PTMs [18]. There are a variety of plants applied in bioprocessing with similar glycosylation systems to eukaryotic cells, including tobacco, potato, and rice, for the production of IF [97]. Type III IF has activity against herpes simplex virus (HSV) and cytomegalovirus (CMV) comparable to type I IFN [98]. High yields of IF can be produced using prokaryotic and eukaryotic expression systems. However, the impact of glycosylation and the short half-life of the IF impacts on clinical use. Modification of IF is carried out using polyethylene glycol (PEG) to improve the half-life; however, PEG can impact the drug safety profile in situ and reduce its activity [97]. Indeed, the majority of therapeutic IFs in use are recombinant proteins expressed in *E. coli* with PEG modification [97].

Monoclonal antibodies (mAb) have potent immune activity including antiviral action, and glycosylation is also important for their biological activity. Viral neutralization, which is the ability of an antibody to bind to and inactivate a virus, is considered the mode of action of mAbs [99]. Antiviral mAbs are typically immunoglobulin Gs (IgGs), which are recognised by Fcy receptors and the complement system, and may stimulate long-term immunity via activation of humoral and cell-mediated immunity [100]. Currently, CHO cells are the main expression system for mAb production due to their effective PTM ability [36]. The yeast *S. cerevisiae*, for example, is prone to hyper mannose glycosylation, which is not suitable for mAb activity and results in unwanted immunogenic reactions in patients [37]. Recently, mAbs have been produced against numerous human viral pathogens, including H5N1 influenza virus, HIV, HSV, CMV, hepatitis C virus (HCV), Ebola, Marburg, SARS, Dengue, rabies, Hendra, Nipah, yellow fever virus, and WNV [100]. Palivizumab was the first antiviral mAb approved by the FDA for prophylaxis of respiratory syncytial virus (RSV) in high-risk infants [101]. mAb against SARS-CoV-2 targeting the spike protein have demonstrated efficacy in vitro [101]. Indeed, there are approximately 70 mAbs in development for treatment of SARS-CoV-2, with four agents granted emergency use authorization by the FDA as antibody cocktails [92]. Yet, despite promising results in animal models, current research shows anti-SARS-CoV-2 mAbs are ineffective against newer Omicron variants and its subvariants [102]. This has since led to the FDA rescinding its authorization of all four approved agents, including casirivimab/imdevimab developed by Regeneron, sotrovimab developed by GSK, and bamlanivimab/etesevimab and bebtelovimab developed by Eli Lilly [103]. The FDA has additionally withdrawn its emergency use authorisation for the COVID-19 antibody drug Evusheld, as of 26 January 2023 [104]. Evusheld is a combination of two long-acting antibodies, tixagevimab and cilgavimab, which is currently manufactured by AstraZenca. The agent still remains authorised for use in the EU for the prevention of COVID-19 in adults and adolescents aged 12 years and older weighing at least 40 kg [105]. Interestingly, the studies of Jaki et al. (2023) investigated the adaptation of SARS-CoV-2 to the mAb cocktail REGN-COV in a patient presenting with hypogammaglobulinemia and requiring a kidney transplant where SARS-CoV-2 adapted via the acquisition of three spike protein mutations [106]. Upon infection, polyclonal antibodies (pAbs) against different epitopes on viral antigens are generated, having neutralising activity against many epitopes [99]. The use of polyclonal antibodies (pAbs) has shown efficacy against pathogens; for example, ZMapp is a pAb against Ebola [107]. ZMapp is a combination of three chimeric IgG monoclonal antibodies which binds to three epitopes on the viral Ebola surface glycoprotein [108]. Resistance issues arise with mAb therapy where viral mutations and the emergence of variant strains impact mAb action in vivo, which may be overcome by use of mAb cocktails [109]. Antibodies can also be used in display technologies as described for whole yeast display vaccines, where single-chain variable antibody fragments and antigen-binding fragments are displayed on the surface to stimulate immune responses post-administration [36,99].

### 2.5. Other Antiviral Intervention Strategies

A continuous systematic exploration of other novel antiviral intervention strategies is also urgently needed. For example, the zinc-finger antiviral protein (ZAP) can inhibit the replication of a myriad of RNA and DNA viruses, including HIV-1, influenza A virus, hepatitis B virus, alphaviruses, filoviruses and retroviruses [110]. ZAP is an interferon-inducible gene, produced by animal and human cells, that preferentially targets viral CpG-rich RNA sequences [111]. The potential of ZAPs continues to elude current research, with studies demonstrating the capability of ZAP to restrict the SARS-CoV-2 pathogen [112]. Many investigations have also demonstrated the potential antiviral activities of various plant-derived phytochemicals, such as flavonoids, polyphenols, alkaloids, carotenoids, quinines, phytoalexins, lignans, polysaccharides, phytosterols and poly-unsaturated fatty acids [113]. Phytochemicals have adopted several mechanisms to inhibit viral replication, being dependant on the compound and target virus. For example, Di Petrillo et al. (2022) summarise the potential use of the flavonoid quercetin as an antiviral, highlighting its ability to inhibit viral neuraminidase, proteases and RNA/DNA polymerases, to modify various viral proteins and to reduce inflammation caused by infection [114]. Indeed, the medical literature abounds with studies of phytochemicals demonstrating antiviral activity against viruses such as HIV, HPV, hepatitis virus, influenza virus, Dengue virus and SARS-CoV-2, to name a few [113,115]. The carotenoid astaxanthin produced by the microalgae *Haemotococcus pluvialis* has anti-inflammatory, immunomodulatory and antioxidative activity in vivo, which may reduce the cytokine storm seen in COVID patients [53]. However, further research is still needed to fully elude the mechanistic properties of phytochemicals to exploit their potential in target-specific drug delivery systems [116]. Microalgae may also offer a suitable expression system to produce therapeutic compounds such as monoclonal antibodies, proteins and vaccines [53]. Recombinant proteins produced by microalgae include E2 protein (swine fever vaccine), D2-CTB fusion protein used in the oral vaccine against *S. aureus* and E7 oncoprotein in HPV vaccines [53]. The studies of Rashidzadeh et al. (2021) describe the use of self-assembling protein-based nanoparticles, which may find application as respiratory viral vaccines. These authors also describe the application of inorganic and metal nanoparticles, e.g., gold, which possess many advantages as potential vaccine modalities; however, research and application investigations remain under study [83]. Interestingly, silver nanoparticles demonstrated efficacy against respiratory viruses in infected mice, with gold nanoparticles reducing influenza HIV and HSV in mice [84]. The research of Rand et al. (2021) described the augmentation of interferon activity by use of defective interfering particles against COVID-19 [117]. In silico rationally designed nano particles are another potential vaccine modality, which are able to self-assemble with improved antigen surface display. Examples include vaccine-designed BG505 SOSIP-I53-50 nanoparticles for HIV, DS-Cav1-I53-50A nanoparticles for Respiratory Syncytial Virus (RSV) [56] and BG505 SOSIP–T33_dn2 nanoparticles for influenza, HIV and RSV [43]. Nanoparticle nasal delivery systems also have many advantages, including lack of enzyme degradation, long retention time, co-delivery with adjuvants and specific targeting of cells [84]. Much research into these approaches is needed to determine pharmacokinetic and pharmacodynamic activity in vivo, with long term studies warranted. Such methods, however, may offer more rapid and effective antiviral techniques once fully established production and administration methods have been developed. The biocompatibility, biodegradability and green synthesis of natural products such as phytochemicals and natural nanoparticle structures offers the many advantages of these modalities. Their ability to replace current approaches, however, is yet to be determined.

**Table 2:** Outlining current and potential expression systems used in the manufacture of interferons and monoclonal antibodies used in clinical treatment of viral infections.

Expression System		Interferon Produced	Monoclonal Antibody Produced	Comments
Bacterial	*E. coli*, *Bacillus subtillis* [33]	IFNα-2a, IFNα-2b, IFNα-2c, IFNα-con-1, INFβ-1b, INFγ-1b [92]	No approved products	Lack of PTMs limits the production of full-length mAbs, though research is ongoing [118]
Fungal	*P. pastoris*, *S. cerevisiae* Filamentous fungi *Aspergillus species*, *Trichoderma reesei* and *Neurospora crassa* [33]	IFNα-2b [97]	No approved products	Glycoengineered yeast can produce interferons and functional full-length mAbs, however extensive clinical research is still required [119]
Plant	Tobacco (*Nicotiana benthamiana)*	IFN-α2b, IFN-γ [97]	Polyclonal antibody of 3 mAB ZMapp (Ebola) [33,120]	Very-large-scale processes for plant production are still in development and require substantial investments [97,120]
Insect	*B. mori*	Chicken IFN-λ [18] (Not approved for use)	No approved products	Extensive research is still necessary to exploit these systems from preclinical applications to clinical trials [121]
Mammalian (animal)	Hamster (CHOs)	No approved products	Casirivimab and Imdevimab mAbs approved for emergency use against COVID-19 [102]	Although CHO cells continue to dominate, there remain inherent limitations in the synthesis and secretion of many complex RTPs for viral treatment [122,123]
	Mouse		Palivizumab (RSV) and Ibalizumab (HIV) [123]	Only approved antiviral mAbs
Mammalian (human)	Leukocytes	IFNα-n3 [92]	No approved products	Although there are still no approved mAbs produced in this system, there are ongoing clinical and preclinical studies being carried out [124]
	Lymphoblastoid cells	IFNα-n1 [92]		
Microalgae	*Chlamydomonas reinhardtii* (*C. reinhardtii*), Phaeodactylum tricornutum, *Dunaliella salina* (*D. salina*) and *Chlorella vulgaris* [50]	No approved products		Offers a green mode of production, affected by low yield.

Abbreviations: mAb—Monoclonal antibodies, PTM—Post Translational Modification, RTP—Recombinant Therapeutic Protein, RSV—Respiratory Syncytial Virus, HIV—Human Immunodeficiency Virus.

## 3. Transitioning from Discrete Batch Operation to Sustainable Integrated Continuous Bioprocessing Requires a Rethink in Viral Inactivation and Clearance Strategies

There is growing interest in revolutionizing the bioprocessing industry, such as by advancing continuous processing over traditional batch manufacturing; however, currently there is a lack of detail on key inline parameters [125,126]. Schofield (2018) intimated that there remain technical and regulatory hurdles to be met in order to implement continuous bioprocessing. These can be met by addressing knowledge gaps in (a) molecule stability; (b) cost competition; (c) breakthrough biopharma companies that want to make their own clinical material; and (d) large pipeline/low current facility capacity. As continuous downstream manufacturing has yet to be elucidated, the transition from batch to continuous includes a number of elements that would reduce cost, including reduction in the size of and number of hold vessels, reduction in equipment, reduced footprint, reduced volume of chromatography sorbents, reduced buffer requirement, reduced consumables and reduction in time to process (ability to process more). In traditional processing, biopharmaceuticals are made in discrete batches where production begins, runs for a finite period and then stops [127]. In contrast, continuous bioprocesses run all the time, or at least for extended periods [126,127]. In terms of new single-use technological solutions to make continuous bioprocessing feasibility for many drug targets and scales, Schofield (2018) noted that “Pall Biotech has launched Cadence™ BioSMB 80 and 350 systems for scalable continuous chromatography, Cadence virus inactivation for the automated semi-continuous operation of the low pH virus inactivation step and in line diafiltration, for continuous buffer exchange and formulation”. Thus, Pall was seen as the first provider to bring a complete end-to-end continuous platform that is scalable; but challenges remain in converting such a platform into a turn-key fully automated solution [125,128].

There are many drivers of continuous bioprocessing, including process intensification, cost and better, more reproducible quality [129,130]. Although upstream processing is advanced in its transition, where chemostat and perfusion reactors are frequently used at the manufacturing scale [131], downstream processing has only recently commenced this transition. Viral clearance strategies are important for the manufacture of safe biologic drug substances and drug products. Moreover, McDonald (2019) noted that “if the biopharma sector want the most out of continuous manufacturing, they must rethink their viral safety strategies”. The biopharma industry has yet to define preferred comprehensive technologies, approaches and protocols for viral safety in continuous processing [127]. While it is appreciated that current strategies work well for batch-mode production; protocols for continuous-model production and equipment preference are yet to be fully defined. The biopharma industry has been challenged by viral contamination since the dawn of the growth of medicine-making cells in bioreactors [127]; this includes potential viral contamination of raw materials, culture media and cell lines used in biopharmaceuticals. Moreover, if finished drugs are virally contaminated, they could infect patients [127]. Ensuring the production of virus-free biopharmaceuticals is more challenging in continuous bioprocessing [99], but continuous production presents benefits including increasing output, reducing cost and reducing waste [127]. Viral safety, testing and clearance/inactivation methods are very important for large-scale production and are seen as expensive regulatory requirements for a new biological products [126,127].

Martins and co-workers (2020) highlighted that continuous inactivation (VI) has received little attention in efforts to realize fully continuous bioprocessing. Implementation of continuous VI must address a specific minimum incubation time (typically 60 min), where the latter reported on the implementation of a packed bed continuous viral inactivation reactor (CVIR) with narrow residence time distribution (RTD) for low pH incubation using two industry-standard model viruses (i.e., xenotrophic murine leukaemia virus and pseudorabies virus). Martins et al. (2020) reported that their combined CVIR with RTD approach achieved low-pH inactivation kinetics where bioprocessing was equivalent to traditional batch operation. This study also builds on other related research [132,133] that emphasized the important role of continuous VI for enabling a complete integrated continuous bio-manufacturing process.

Thus, the biopharma industry is committed to advancing continuous bioprocessing that reflects market trends and the need to access therapeutics solutions [99]. There will be a commensurate evolution reflecting greater speed and higher quality, which are important for therapeutic proteins. To address these opportunities, new facilities are being designed that further support perfusion bioreactors and continuous downstream solutions, such as multi-column capture and flow-through polishing operations. Convergence of innovation in analytical technologies and advances in other digital technologies (such as sensors and adaptive process controls) will help meet the future potential of continuous bioprocessing [109,134]. Such future developments will inform efficient management of facilities and footprint reduction. Advancing inline viral inactivation presents an important activity to enable future efficacy of continuous bioprocessing as an integral step between capture chromatography and flow-through polishing [134]. Additional emphasis will be placed on validating inline viral inactivation parameters for regulatory system scrutiny informed by small-scale virus-spiking studies for mAb continuous production [134].

## 4. Conclusions

Antiviral biologics are vitally important to curb the impact of emerging viral infectious disease and reduce the risk of pandemic-scale events. Challenges relating to vaccine development include the highly divergent nature of viral species which are prone to mutagenic events and the development of variants resistant to vaccines. The host response to vaccination is also variable and influenced by age, gender, co-morbidities, pre-existing immune status and nutritional aspects. The recent COVID-19 pandemic and the limitations of traditional vaccine production systems have encouraged the development of novel vaccine methods including viral particles, yeast-based deliver systems and nucleic acid vaccines, which are faster to produce and more amenable to variant strains. Undoubtedly, recombinant DNA technology, genetic engineering and large-scale bioprocessing has aided in recent antiviral development strategies [134]. Additional therapeutic options such as interferons and monoclonal antibodies are also in development as treatment options where small molecule therapy is currently the mainstay. Transitioning from discrete batch operation to sustainable integrated continuous bioprocessing requires a rethink in viral inactivation and clearance strategies.

## Figures and Tables

**Table 1:** Outline of current and potential expression systems used in the manufacture of vaccines, and their advantages and limitations.

Expression System		Advantages	Limitations	Vaccine Produced
Bacterial	*E. coli* *Pseudomonas fluorescens*, *Ralstonia eutropha*, *Bacillus or Lactococcus* species are possible alternatives to *E. coli* [42]	Simple structure, rapid growth rate, high product yield, easy genetic manipulations, low cost, scalable [43]	Inability to perform PTMs, expression of misfolded, insoluble, or non-functional proteins, endotoxin contamination [43]	Hepatitis E, human papillomavirus, and meningococcal vaccines [43,44]
Fungal	*S. cerevisiae*	Rapid growth rate, high product yield, easy genetic manipulations, secretory expression, low cost and scalable, capacity to perform PTMs [43]	Low yields of protein expression, hyperglycosylation [43]	Hepatitis B and human papillomavirus vaccines [43]
	*P. pastoris* *Yarrowia lipolitica*, *Arxula adeninivorans* and *Kluyveromyces lactis* [42] Filamentous fungi *Aspergillus* and *Trichoderma* [33]		Glycosylation differs to mammalian cells Large volumes of methanol required [45]	Hepatitis B vaccine [45] Infectious bursal disease (IBD) in poultry [42] IFN alpha 2, IL-6 [42]
Plant	*Nicotiana benthamiana* *Transgenic plants*, e.g., *Lemna duckweed*	Cost effective production high product yield, reduced contamination risk, capacity to perform PTMS, oral administration, scalable [46]	Lack of regulation and GMP, glycosylation differs to mammalian cells [47]	COVID-19 (Covifenz^®^) vaccine [48]
Insect	*Spodoptera frugiperda* *Baculovirus expression vector system* (*BEVS*), Spodoptera frugiperda, rosophila *Schneider line 2* (*S2 cells*) [43]	High product yield, capacity to perform PTMs, BEVS increases expression levels and safety [43]	Demand higher costs, more laborious, difficult to scale-up, glycosylation differs to mammalian cells [43]	Human papillomavirus, and influenza vaccines [43]
Mammalian (animal)	Hamster (CHOs)	Capacity to perform complex human-like PTMs, high product yield, scalable, well-established regulatory track record [43]	Slow production speed, expensive, contamination with animal viruses, produce PTMs not expressed in humans, i.e., α-gal and NGNA [43,49]	Herpes zoster vaccine [43]
	Monkey (VERO)			Influenza, polio, rabies and Ebola virus vaccines [50]
Mammalian (human)	HEK293	Capacity to perform complex, fully human PTMs, easy to reproduce, maintain, manipulate and transiently transfect [49]	Potential for human-specific viral contamination, lack of extensive clinical experience compared to other cell lines [51]	COVID-19 (Ad5-nCOV and ChAdOX1-nCoV) vaccine [52]
Transgenic animals	Goat milk, cow milk, hens (embryo) [42]	Large yield, PTMs	Ethical issues	Human recombinant albumin, insulin [42] not established for vaccine production
Microalgae	Chlamydomonas reinhardtii (*C. reinhardtii*), Phaeodactylum tricornutum, Dunaliella salina (*D. salina*) and *Chlorella vulgaris* and non-photosynthetic microalgae such as *Schizochytrium* sp. [43].	Including rapid transformation, high growth rate, ease of growth, low cost, PTMs, absence of toxin compounds	Low expression, improper PTMs	Good potential for oral vaccine delivery, malaria, HPV, Zika are currently investigated [43], viral protein 28 (VP 28) [53]

Abbreviations: PTMs—Post Translation Modifications, CHO—Chinese Hamster Ovary, HEK293—Human Embryonic Kidney, BEVS—Baculovirus Expression Vector System, α-gal—galactose-α1,3-galactose, NGNA—*N*-glycolylneuraminic acid.

## Data Availability

Data sharing not applicable.
